# Survey data regarding perceived air quality in Australia, Brazil, China, Ghana, India, Iran, Italy, Norway, South Africa, United States before and during Covid-19 restrictions

**DOI:** 10.1016/j.dib.2020.106169

**Published:** 2020-08-13

**Authors:** Diego Maria Barbieri, Baowen Lou, Marco Passavanti, Cang Hui, Daniela Antunes Lessa, Brij Maharaj, Arunabha Banerjee, Fusong Wang, Kevin Chang, Bhaven Naik, Lei Yu, Zhuangzhuang Liu, Gaurav Sikka, Andrew Tucker, Ali Foroutan Mirhosseini, Sahra Naseri, Yaning Qiao, Akshay Gupta, Montasir Abbas, Kevin Fang, Navid Ghasemi, Prince Peprah, Shubham Goswami, Amir Hessami, Nithin Agarwal, Louisa Lam, Solomon Adomako

**Affiliations:** a Norwegian University of Science and Technology, Department of Civil and Environmental Engineering. Høgskoleringen 7A, Trondheim, 7491, Trøndelag, Norway; b Chang'an University, School of Highway, Nan Er Huan Road (Mid-section), Xi'an, 710064, Shaanxi, China; c Italian Society of Cognitive Behavioural Therapy (CBT-Italy), Guastalla St. 2, Carpi 4012, Emilia-Romagna, Italy; d Centre for Invasion Biology, Department of Mathematical Sciences, Stellenbosch University, Matieland, 7602, South Africa.; e Federal University of Ouro Preto, Department of Civil Engineering. Rua Nove, Bauxita, Ouro Preto, 35400-000, Minas Gerais, Brazil; f University of KwaZulu-Natal, Department of Geography. Howard College City, Durban, 4000, KwaZulu, South Africa; g Indian Institute of Technology Guwahati, Department of Civil Engineering. IIT Guwahati, Guwahati, 781039, Assam, India; h State Key Laboratory of Silicate Materials for Architectures, Wuhan University of Technology. Luoshi road 122, Wuhan, 430070, Hubei, China; i University of Idaho, Department of Civil and Environmental Engineering. 875 Perimeter Drive, Mailstop 1022, Moscow, 83844, Idaho, United States; j Ohio University, Department of Civil Engineering/Russ College of Engineering & Technology. 28 W. Green Drive, Athens, 45701, Ohio, United States; k Sun Yat-sen University, School of Civil Engineering. Xingang Xi Road 135, Guangzhou, 510275, Guangdong, China; l Chang'an University, School of Highway. Nan Er Huan Road (Mid-section), Xi'an, 710064, Shaanxi, China; m Lalit Narayan Mithila University, Department of Geography. Darbhanga, 846004, Bihar, India; n University of Connecticut, Connecticut Transportation Safety Research Center. 270 Middle Turnpike, Unit 5202 Longley Building, Storrs, 06269, Connecticut, United States; o Norwegian University of Science and Technology, Department of Civil and Environmental Engineering. Høgskoleringen 7A, Trondheim, 7491, Trøndelag, Norway; p Bam University of Medical Sciences, School of Medicine. Bam, 76615-336, Kerman, Iran; q China University of Mining and Technology, School of Mechanics and Civil Engineering. Daxue Road 1, Xuzhou, 22116, Jiangsu, China; r Indian Institute of Technology Roorkee, Department of Civil Engineering, Transportation Engineering Group, 321-A&B, Roorkee, 247667, Uttarakhand, India; s Virginia Tech, Department of Civil and Environmental Engineering. 301-D3 Patton Hall, Blacksburg, 24061, Virginia, United States; t Sonoma State University, Department of Geography, Environment, and Planning, 1801 East Cotati Avenue, Rohnert Park, 94928, California, United States; u University of Bologna, Department of Civil Chemical Environmental and Materials Engineering. Viale del Risorgimento, 2, Bologna, 40136, Emilia-Romagna, Italy; v University of New South Wales, Department of Social Policy Research Centre, John Goodsell Building, Kensington, Sydney, 2052, New South Wales, Australia; w Indian Institute of Science Bangalore, Department of Civil Engineering, C V Raman Avenue, Bangalore, 560012, Karnataka, India; x Texas A&M University – Kingsville, Department of Civil and Architectural Engineering, 917 W. Ave B, Kingsville, 78363, Texas, United States; y University of Florida, Department of Civil & Coastal Engineering, 2100 NE Waldo Rd., Sta 106, Gainesville, 32609, Florida, United States; z Federation University Australia, School of Nursing and Healthcare Professions, 72-100 Clyde Rd, Berwick, 3806, Victoria, Australia; $ University of Agder, Department of Engineering and Science, Jon Lilletuns vei 9, Grimstad, 4879, Agder, Norway

**Keywords:** Survey data, COVID-19, Environmental pollution, Air quality, Psychometric perception

## Abstract

The dataset deals with the air quality perceived by citizens before and during the enforcement of COVID-19 restrictions in ten countries around the world: Australia, Brazil, China, Ghana, India, Iran, Italy, Norway, South Africa and the United States. An online survey conveniently translated into Chinese, English, Italian, Norwegian, Persian, Portuguese collected information regarding the perceived quality of air pollution according to a Likert scale. The questionnaire was distributed between 11-05-2020 and 31-05-2020 and 9 394 respondents took part. Both the survey and the dataset (stored in a Microsoft Excel Worksheet) are available in a public repository. The collected data offer the people's subjective perspectives related to the objective improvement in air quality occurred during the COVID-19 restrictions. Furthermore, the dataset can be used for research studies involving the reduction in air pollution as experienced, to a different extent, by populations of all the ten countries.

**Specification table**

**Table:** 

Subject	Social Sciences
Specific subject area	Health psychology, Perceived air pollution
Type of data	Primary data, Table
How data were acquired	The data were collected by an online survey hosted on two platforms: Google Forms (English, Italian, Norwegian, Persian, Portuguese versions) and WenJuanXing (Chinese version). An English copy is available in the data repository. The survey was distributed by means of professional and social networks
Data format	Raw Analyzed
Parameters for data collection	The survey data were obtained from 9 394 respondents older than 18 years old having internet access
Description of data collection	The online survey was distributed using a combination of purposive and snowball techniques
Data source location	Countries: Australia, Brazil, China, Ghana, India, Iran, Italy, Norway, South Africa and the United States
Data accessibility	Dataset is uploaded on Mendeley Data Repository name: Perceived air pollution in Australia, Brazil, China, Ghana, India, Iran, Italy, Norway, South Africa, USA before and during COVID-19 restrictions Data identification number: DOI: 10.17632/fb38h4tyzn.2 Direct URL to data: https://data.mendeley.com/datasets/fb38h4tyzn/2

Value of the data

• The data are related to the perception of air quality and air pollution during the COVID-19 restrictions as experienced by a large pool comprising 9 394 respondents located in ten countries on six continents
• The data can be useful for researchers dealing with the environmental and tropospheric changes occurring during the COVID-19 restrictions
• The data can be used to assess the relationship between the perceived and the quantified change in air quality and air pollution during the COVID-19 restrictions
• The data can be of interest to both citizens and policymakers to realise the tremendous lesson learned during COVID-19, being air quality a key indicator for sustainable development

## 1 Data description

**Fig. 1:**
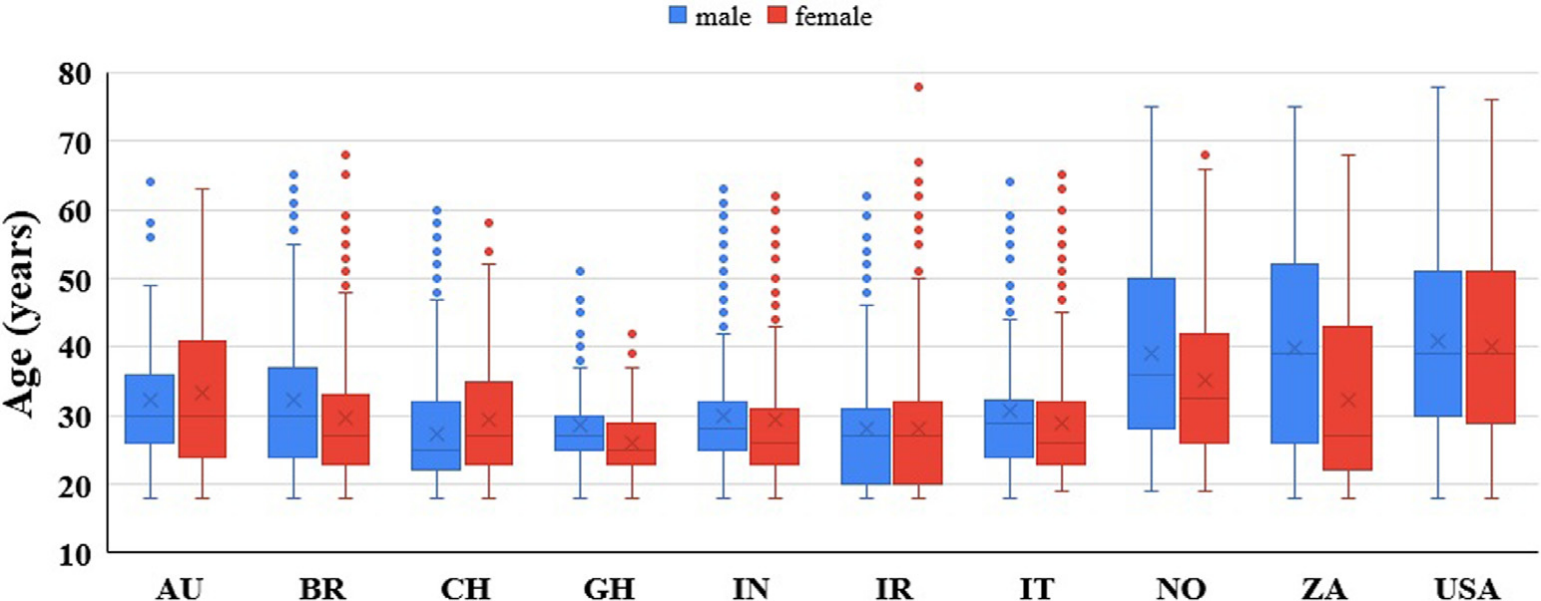
Age and gender of the respondents for each country.

**Fig. 2:**
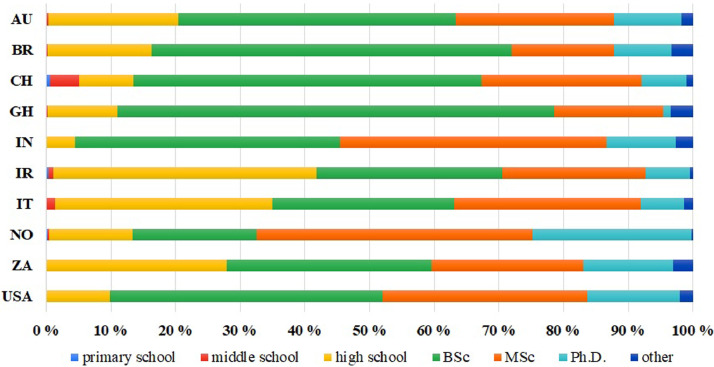
Education of the respondents for each country.

**Fig. 3:**
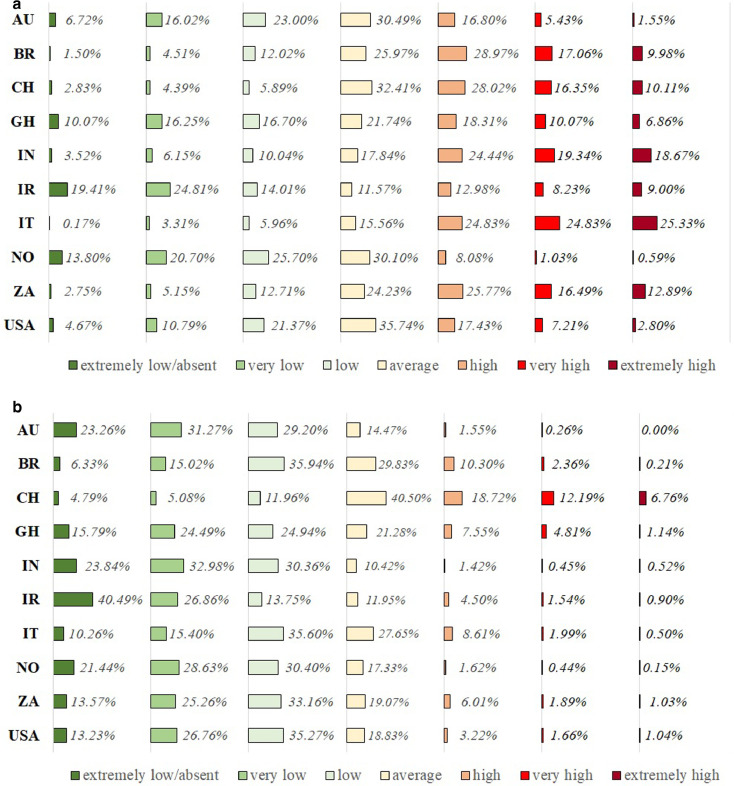
Perceived amount of air pollution before (a) and during (b) the COVID-19 restrictions as experienced by the survey respondents in each country.

The dataset provides information regarding the quantity of air pollution perceived before and during the restrictions enforced in ten countries around the world as a consequence of the COVID-19 pandemic: Australia, Brazil, China, Ghana, India, Iran, Italy, Norway, South Africa and the United States (also referred to as AU, BR, CH, GH, IN, IR, IT, NO, ZA and USA, respectively). The dataset is stored in a public repository as Microsoft Excel Worksheet [1]. The total amount of the respondents who joined the survey is 9 394, their geographical distribution is reported in Table 1. Information regarding gender and age are reported in Fig. 1 with box-and-whisker plots: overall, the largest portion of the surveyed population is composed of young and middle-aged individuals. Furthermore, the participants have high education (Fig. 2). The two questions of the survey are “How do you regard the amount of air pollution before the epidemic?” and “How do you regard the amount of air pollution during the restrictions?”: the respondents expressed their opinions according to a 7-point Likert scale varying from “extremely low/absent air pollution” to “extremely high air pollution”. The responses pertaining to before and during the applications of the COVID-19 restrictions are reported in Fig. 3a and Fig. 3b, respectively.

**Table 1:** Geographical distribution of survey respondents.

AUSTRALIA - AU (N = 387)			
Victoria	New South Wales	Queensland	South Australia
40.6 %	29.2 %	16.3 %	11.9 %
Western Australia	Tasmania	Northern Territory	Australian Capital Territory
0.8 %	0.5 %	0.5 %	0.3 %
BRAZIL - BR (N = 930)			
Minas Gerais	São Paulo	Rio de Janeiro	Bahia
60.0 %	21.6 %	3.7 %	2.4 %
Distrito Federal	Santa Catarina	Paraná	Espírito Santo
2.3 %	1.7 %	1.3 %	1.1 %
Goiás	Mato Grosso	Rio Grande do Sul	Pernambuco
1.0 %	1.0 %	0.9 %	0.5 %
Rio Grande do Norte	Alagoas	Pará	Amazonas
0.5 %	0.4 %	0.4 %	0.3 %
Mato Grosso do Sul	Paraíba	Tocantins	Ceará
0.3 %	0.2 %	0.2 %	0.1 %
Piauí	*other*		
0.1 %	0.0 %		
CHINA - CH (N = 1731)			
Guangdong	Shaanxi	Jiangsu	Hunan
14.9 %	13.1 %	11.9 %	6.9 %
Anhui	Gansu	Hebei	Hubei
4.9 %	4.7 %	4.2 %	3.8 %
Shandong	Beijing	Shanxi	Heilongjiang
3.6 %	3.5 %	3.0 %	2.7 %
Sichuan	Henan	Inner Mongolia	Fujian
2.0 %	1.8 %	1.8 %	1.7 %
Jiangxi	Guangxi	Tianjin	Hainan
1.6 %	1.3 %	1.2 %	1.1 %
Jilin	Chongqing	Liaoning	Guizhou
1.1 %	1.0 %	1.0 %	1.0 %
Shanghai	Xinjiang	Ningxia	Zhejiang
1.0 %	0.9 %	0.9 %	0.8 %
Qinghai	Yunnan	Taiwan	Tibet
0.6 %	0.5 %	0.5 %	0.5 %
Macau	Hong Kong		
0.4 %	0.3 %		
GHANA - GH (N = 437)			
Greater Accra	Ashanti	Northern	Eastern
29.7 %	27.0 %	10.3 %	8.5 %
Central	Western Region	Volta Region	Bono Region
6.4 %	5.0 %	3.4 %	2.1 %
Upper East	Bono East Region	Upper West	Ahafo Region
2.1 %	1.6 %	1.6 %	1.1%
Oti	Savannah	North East	Western North
0.5 %	0.2 %	0.2%	0.2%
INDIA - IN (N = 1334)			
West Bengal	Maharashtra	NCR Delhi	Rajasthan
15.0 %	13.2 %	9.2 %	7.4 %
Uttar Pradesh	Tamil Nadu	Karnataka	Bihar
6.8 %	6.7 %	6.7 %	6.6 %
Madhya Pradesh	Haryana	Uttarakhand	Gujarat
4.9 %	3.9 %	3.7 %	2.8 %
Assam	Telangana	Punjab	Jammu & Kashmir
2.0 %	1.7 %	1.6 %	1.3 %
Andhra Pradesh	Odisha	Himachal Pradesh	Kerala
1.2 %	0.9 %	0.8 %	0.8 %
Goa	Jharkhand	Chhattisgarh	Meghalaya
0.7 %	0.7 %	0.4 %	0.3 %
Chandigarh	Ladakh	Puducherry	Tripura
0.1 %	0.1 %	0.1 %	0.1 %
*other*			
0.0 %			
IRAN - IR (N = 778)			
Kerman	Tehran	Fars	Razavi Khorasan
48.7 %	28.5 %	5.1 %	5.0 %
Isfahan	Yazd	Mazandaran	East Azarbaijan
3.3 %	1.5 %	1.4 %	1.2 %
Alborz	Hormozgan	Hamedan	West Azerbaijan
0.8 %	0.6%	0.6 %	0.5 %
Qazvin	Sistan Baluchestan	Kermanshah	Kohg. B.-Ahmad
0.5 %	0.4 %	0.4 %	0.3%
Golestan	Ilam	Bushehr	North Khorasan
0.3 %	0.1 %	0.1 %	0.1 %
South Khorasan	Zanjan	Semnan	*other*
0.1 %	0.1 %	0.1 %	0.0 %
ITALY - IT (N = 604)			
Emilia-Romagna	Lombardiao	Lazio	Veneto
32.5 %	17.7 %	12.1 %	9.8 %
Piemonte	Toscana	Campania	Puglia
8.8 %	3.6 %	2.5 %	2.3 %
Friuli-Venezia Giulia	Sicilia	Marche	Calabria
2.2 %	1.7 %	1.3 %	1.2 %
Liguria	Sardegna	Trentino-Alto Adige	Abruzzo
1.0 %	0.8 %	0.8 %	0.5 %
Molise	Umbria	Valle d'Aosta	*other*
0.5 %	0.5%	0.3%	0.0 %
NORWAY - NO (N = 681)			
Trøndelag	Rogaland	Oslo	Viken
54.2 %	13.4 %	9.0%	5.9 %
Agder	Innlandet	Møre og Romsdal	Vestland
5.4 %	5.0 %	2.8 %	1.9%
Troms og Finnmark	Vestfold og Telemark	*other*	
1.6 %	0.9 %	0.0 %	
SOUTH AFRICA - ZA (N = 582)			
KwaZulu-Natal	Gauteng	Western Cape	Eastern Cape
61.7 %	16.0%	10.5%	6.4 %
North West	Mpumalanga	Free State	Limpopo
2.4 %	1.2 %	1.0%	0.9 %
*other*			
0.0 %			
UNITED STATES - USA (N = 1928)			
Connecticut	Ohio	Texas	California
13.9 %	13.6 %	12.7 %	11.3 %
Idaho	Florida	Virginia	Washington
6.9 %	6.8 %	6.7 %	5.9 %
North Carolina	Illinois	Arizona	New York
2.7 %	2.1 %	1.3 %	1.3 %
Colorado	Oregon	Pennsylvania	Michigan
1.2 %	1.2 %	1.1 %	1.0 %
Massachusetts	New Jersey	Wisconsin	Georgia
1.0 %	1.0 %	0.6 %	0.6 %
Maryland	Vermont	Indiana	Iowa
0.5 %	0.5 %	0.4 %	0.4 %
Nevada	South Carolina	Minnesota	Missouri
0.4 %	0.4 %	0.4 %	0.4 %
Tennessee	Kentucky	Washington D.C. Columbia	Alaska
0.4 %	0.3 %	0.3 %	0.3 %
West Virginia	Alabama	Arkansas	Kansas
0.3 %	0.2 %	0.2 %	0.2 %
Louisiana	New Hampshire	Montana	North Dakota
0.2 %	0.2 %	0.2 %	0.1 %
Maine	Rhode Island	Wyoming	Hawaii
0.1 %	0.1 %	0.1 %	0.1 %
Nebraska	New Mexico	Oklahoma	South Dakota
0.1 %	0.1 %	0.1 %	0.1 %
Utah	Guam	US Virgin Islands	*other*
0.1 %	0.1 %	0.1 %	0.0 %

## 2 Experimental design, materials, and methods

The online survey has assessed the air quality as subjectively perceived by citizens in ten countries: Australia, Brazil, China, Ghana, India, Iran, Italy, Norway, South Africa and the United States. The online questionnaire was hosted on two platforms: Google Forms (English, Italian, Norwegian, Persian, Portuguese versions) and WenJuanXing (Chinese version) and promoted on professional and social networks. The survey content was the same for each language; only the question regarding the respondents’ geographical location was tailored for each country. A Likert scale was employed to collect information about subjective perceptions [2] regarding both the situation before and during the enforcement of the restrictions due to the COVID-19 pandemic [3,4]. The online survey was distributed using a combination of purposive and snowball techniques between 11-05-2020 and 31-05-2020. Previously, other opinion surveys at regional and national scale also dealt with the perception of air quality [5], [6], [7] and examined the psychological impacts on people's subjective emotional state [8]. The created dataset can allow to explore how air quality was experienced by the populations dealing with different levels of air pollution before the COVID-19 outbreak [9], [10], [11].

## Ethics statement

All the survey respondents informed their consent before joining the survey consistent with the Declaration of Helsinki.

## Credit Author Statement

Diego Maria Barbieri

Conceptualization, Methodology, Formal analysis, Investigation, Resources, Data curation, Writing - Original Draft, Visualization, Project administration

Baowen Lou

Conceptualization, Methodology, Formal analysis, Investigation, Resources, Data curation, Writing - Original Draft, Visualization

Marco Passavanti

Conceptualization, Methodology, Investigation, Writing - Original Draft, Visualization

Cang Hui

Investigation, Data curation, Writing - Review & Editing, Visualization, Supervision

Daniela Antunes Lessa

Investigation, Data curation

Brij Maharaj

Investigation, Data curation

Arunabha Banerjee

Investigation, Data curation

Fusong Wang

Investigation, Data curation

Kevin Chang

Investigation, Data curation

Bhaven Naik

Investigation, Data curation

Lei Yu

Investigation, Data curation

Zhuangzhuang Liu

Investigation, Data curation

Gaurav Sikka

Investigation, Data curation

Andrew Tucker

Investigation, Data curation

Ali Foroutan Mirhosseini

Investigation, Data curation

Sahra Naseri

Investigation, Data curation

Yaning Qiao

Investigation, Data curation

Akshay Gupta

Investigation, Data curation

Montasir Abbas

Investigation, Data curation

Kevin Fang

Investigation, Data curation

Navid Ghasemi

Investigation, Data curation

Prince Peprah

Investigation, Data curation

Shubham Goswami

Investigation, Data curation

Amir Hessami

Investigation, Data curation

Nithin Agarwal

Investigation, Data curation

Louisa Lam

Investigation, Data curation

Solomon Adomako

Investigation, Data curation

## Declaration of competing interest

This research has not received any specific grant from funding agencies in the public, commercial, or not-for-profit sectors.

## Appendix Supplementary materials

Image, application 1
